# Diagnostic workup in paradoxical urticaria induced by H1‐antihistamines

**DOI:** 10.1111/jdv.20523

**Published:** 2025-03-25

**Authors:** Yi‐Kui Xiang, Emek Kocatürk

**Affiliations:** ^1^ Institute of Allergology Charité – Universitätsmedizin Berlin, Corporate Member of Freie Universität Berlin and Humboldt‐Universität Zu Berlin Berlin Germany; ^2^ Fraunhofer Institute for Translational Medicine and Pharmacology ITMP, Immunology and Allergology Berlin Germany; ^3^ Shanghai Skin Disease Hospital Tongji University School of Medicine Shanghai China; ^4^ Department of Dermatology Bahcesehir University School of Medicine Istanbul Turkey

The recent study published by Soria et al.^1^ in JEADV effectively summarizes 16 rare cases of paradoxical urticaria (PU) to H1‐antihistamines (H1A), highlighting the complexities involved when urticaria cases respond atypically to H1A treatment. The authors stress the knowledge gaps in clinical features of PU as well as its mechanisms, and the need for further research to guide dermatology and allergy specialists.

H1‐antihistamines are the recommended cornerstone treatment for all forms of urticaria.^2^ However, it is noteworthy that some urticaria cases are paradoxically triggered by H1A themselves, a phenomenon termed PU. Diagnosing PU presents a significant challenge, particularly in patients with pre‐existing chronic spontaneous urticaria (CSU), as it is difficult to discern whether the flare of wheals and/or angioedema are attributable to an exacerbation of the underlying CSU or to a reaction to H1A. To address this, the study proposes diagnostic criteria: the occurrence of hives at least with two different classes of H1A within 5 h of intake, as all of the 16 reported cases had reactions with ≥2 different H1A and the onset occurred mostly within 2–5 h. This timing criterion may not be entirely precise, and further studies with larger cohorts and more detailed documentation of PU cases are needed to refine and validate it.

Another challenge in diagnosing PU is the need to rule out H1A‐induced anaphylaxis or immediate hypersensitivity presenting with urticarial symptoms. This is accomplished through negative skin testing, including prick tests, intradermal testing and patch tests. This is based on the finding that most reported cases of H1A‐induced anaphylaxis or immediate hypersensitivity present with urticaria have positive skin tests.^3^, ^4^ As part of the diagnostic workup for PU, skin testing should also be conducted on the drugs selected for oral provocation testing or oral substitution testing prior to their administration.

Despite the rarity of PU and the limited reports and evidence elucidating the mechanisms by how H1A trigger PU, certain clinical characteristics can still be observed. PU predominantly occurs in patients with CSU, often in those with an atopic background, additionally, a subset of these patients exhibits cross‐intolerance to NSAIDs, raising the question of whether PU may be associated with Type 1 autoallergic CSU where an association with NSAID intolerance has been reported.^5^ Further studies should investigate immunological parameters including levels of total IgE, thyroid antibodies and especially autoreactive IgE in patients with PU to better understand its underlying mechanisms and potential associations with CSU endotypes.^6^

PUs occurred almost with every class and type of H1As including cetirizine, desloratadine, chlorpheniramine, ebastine, olapataine, fexofenadine, hydroxyzine, dexchlorpheniramine and loratadine, but how do they trigger PU? Several hypotheses have been proposed. First, non‐IgE‐mediated mast cell activation pathways such as the complement pathway, G‐protein–coupled receptors other than histamine receptors, such as MRGPRX2 on mast cell membranes, might play a role. Second, similar to NSAIDs, H1As exhibit anti‐inflammatory properties. Mechanisms underlying NSAID hypersensitivity, such as prostaglandin inhibition and relative overproduction of leukotrienes, could potentially be applied to PU pathogenesis. Lastly, and perhaps most intriguingly, is the hypothesis that H1As themselves act as haptens or that their binding to H1 receptors induces an active conformational change. Additionally, the chemical structure of H1As, such as the ethylamine group, may share epitope similarities with histamine.

In conclusion, the study by Soria et al.^1^ provides a diagnostic workup (Figure 1) for paradoxical urticaria induced by H1A, while also highlighting critical knowledge gaps that require further investigation. This study underscores the importance of recognizing that even ‘anti‐urticaria’ medications can paradoxically induce urticaria. In such instances, a comprehensive diagnostic workup is crucial to prevent delays in addressing the underlying condition and to minimize the risk of additional complications.

**FIGURE 1:**
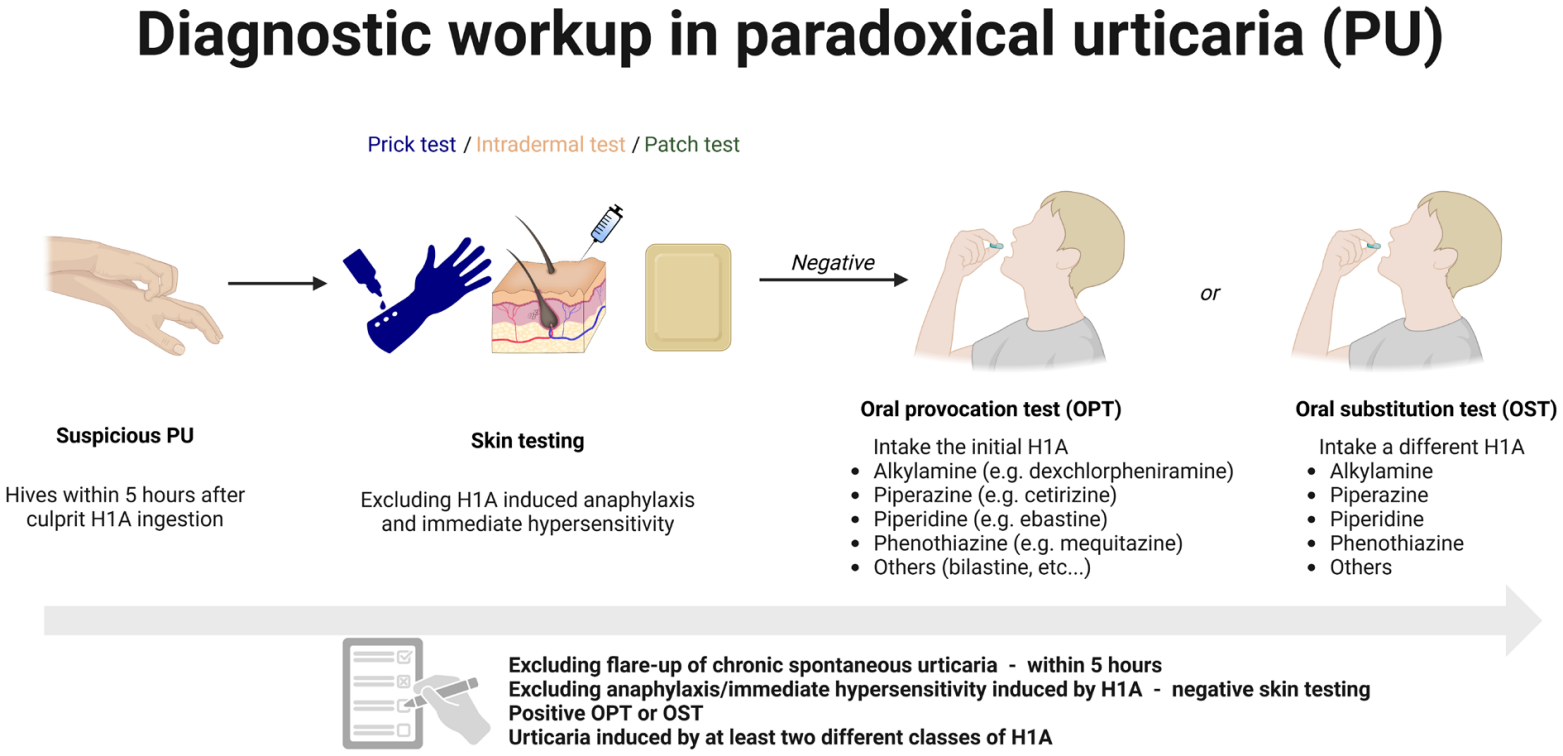
Diagnostic workup in PU includes urticaria occurrence within 5 h after H1A ingestion (with at least two different classes) together with negative skin testing and positive oral test.

## ACKNOWLEDGEMENTS

Open Access funding enabled and organized by Projekt DEAL.

## CONFLICT OF INTEREST STATEMENT

YX has no conflicts of interest to declare. EK has been a speaker/advisor for Novartis and Menarini.

## Data Availability

Data sharing not applicable to this article as no datasets were generated or analysed during the current study.
